# Pan-cancer analysis: predictive role of TAP1 in cancer prognosis and response to immunotherapy

**DOI:** 10.1186/s12885-022-10491-w

**Published:** 2023-02-09

**Authors:** Zewei Tu, Kuangxun Li, Qiankun Ji, Yuyang Huang, Shigang Lv, Jingying Li, Lei Wu, Kai Huang, Xingen Zhu

**Affiliations:** 1 Department of Neurosurgery, The Second Affiliated Hospital of Nanchang University, Jiangxi 330006 Nanchang, P. R. China; 2 Jiangxi Key Laboratory of Neurological Tumors and Cerebrovascular Diseases, Jiangxi 330006 Nanchang, P. R. China; 3 Institute of Neuroscience, Nanchang University, Jiangxi 330006 Nanchang, P. R. China; 4 JXHC Key Laboratory of Neurological Medicine, Jiangxi 330006 Nanchang, P. R. China; 5 Queen Mary School, University of Nanchang, Jiangxi 330006 Nanchang, P. R. China; 6 Department of Comprehensive Intensive Care Unit, The Second Affiliated Hospital of Nanchang University, Nanchang, P. R. China

**Keywords:** Transporter associated with antigen processing 1 (TAP1), Pan-cancer, Prognostic biomarker, Cancer immunotherapy, Immune Check-point Inhibitor (ICI)

## Abstract

**Background:**

Transporter associated with antigen processing 1 (TAP1) is a molecule involved in processing and presentation of major histocompatibility complex class I restricted antigens, including tumor-associated antigens. TAP1 participates in tumor immunity, and is aberrantly expressed in multiple cancer types;

**Methods:**

Transcriptome profiles were obtained from The Cancer Genome Atlas and Genotype-Tissue Expression databases. Genetic alterations, protein distribution, and interaction information for TAP1 were downloaded from cBioPortal, Human Protein Atlas and Compartmentalized Protein–Protein Interaction, respectively. Single-cell analyses of TAP1 across cancers were conducted via the Tumor Immune Single-cell Hub website. Gene set enrichment analysis was employed to investigate TAP1-associated functional mechanisms and processes. Immune cell infiltration was explored using Tumor Immune Estimation Resource 2.0. Pan-cancer correlations between *TAP1* expression and immunotherapy biomarkers were explored using the Spearman’s correlation test. Associations with immunotherapy responses were also investigated using clinicopathological and prognostic information from cohorts of patients with cancer receiving immune checkpoint inhibitors.

**Results:**

*TAP1* expression was elevated in most cancer types and exhibited distinct prognostic value. Immune cells expressed more *TAP1* than malignant cells within most tumors. *TAP1* expression was significantly correlated with immune-related pathways, T-lymphocyte infiltration, and immunotherapeutic biomarkers. Clinical cohort validation revealed a significant correlation with immune therapeutic effects and verified the prognostic role of TAP1 in immunotherapy. Western blot assay indicated that TAP1 is upregulated in glioblastoma compared with adjacent normal brain tissues.

**Conclusion:**

TAP1 is a robust tumor prognostic biomarker and a novel predictor of clinical prognosis and immunotherapeutic responses in various cancer types.

**Supplementary Information:**

The online version contains supplementary material available at 10.1186/s12885-022-10491-w.

## Background

Transporter associated with antigen processing 1 (TAP1) is a member of the ATP-binding cassette superfamily, which forms a heterodimeric complex with its homolog, TAP2, for intracellular translocation of antigenic peptide across endoplasmic reticulum (ER) membrane [1, 2]. In the ER, TAP complexes assist in loading cytosolic peptides onto adjacent major histocompatibility complex class I (MHC-I) molecules, which then transport the peptide to the cell surface for recognition by CD8^+^ cytotoxic T lymphocytes (CTL). MHC-I presentation occurs in every nucleated cell (i.e., not mature erythrocytes), and presented antigens are generally derived from endogenous molecules [3]; however, MHC-I molecules in dendritic cells (DCs) can also present exogenous antigens derived from pathogens or dead cells [4]. Due to precise regulation, viral-infected cells and malignantly transformed cells that express abnormal proteins are under strict immune surveillance and are eliminated over time. Consequently, the pivotal function of TAP1 is prone to be hijacked for immune evasion by malignant disease.

The TAP complex executes its role via an elaborate mechanism. Antigenic proteins, either endogenously expressed or internalized by antigen presenting cells, are marked by ubiquitin and degraded into small, 8–10 amino acid, peptide fragments [5]. CD8^+^ CTL recognize the antigenic peptides presented by MHC-I and initiate an immune attack. When errors in assembly or translocation of MHC-I complexes occur, CTL-mediated immune surveillance is suppressed [2]. Therefore, malignant cells evolve strategies to escape immune system recognition by targeting and interfering with the normal process of MHC-I-mediated antigen presentation, particularly the “peptide pump”, TAP [6]. Within the TAP complex, TAP1 stabilizes the assembly of TAP2 [7]. Thus, we focused on TAP1 as potentially dominant in TAP protein function. Studies on TAP1 have continuously emerged over recent years, with novel findings in several cancers [8]. Down-regulation or defective TAP1 expression were observed in primary cancer or autologous metastatic lesions of different disease stages, including in bladder cancer [9], small cell lung cancer [10], glioma [11], prostatic cancer [12], head and neck squamous cell carcinoma (HNSC) [13], breast cancer [14], and colorectal cancer (CRC) [15]. However, few studies have proposed associations between TAP1 and responses to therapeutic anti-tumor regimens. As a novel treatment approach, immune therapy delays, or even completely blocks, tumor development and progression, providing hope for many cancer patients. Nevertheless, expected responses to immunotherapy are not observed in every patient. Further, random application of immunotherapy can be disadvantageous to patients who exhibit tolerance or toxic reactions to the drugs. Therefore, exploring reliable biomarkers that can predict the effect of immunotherapy for individual patients is an urgent priority. TAP1 regulates normal immune responses, and is abnormally expressed in various cancer types; therefore, we hypothesized that it is a potential biomarker for predicting immunotherapeutic efficacy.

In this study, we performed a comprehensive pan-cancer analysis to generate a *TAP1* expression landscape across various cancer types. Further, we report basic information regarding TAP1 in pan-cancer cohorts and explore the relationship between *TAP1* expression and prognosis, enriched gene sets, immune cell infiltration, expression of immune regulators, and immunotherapeutic effects on a pan-cancer scale. Based on these data, we propose TAP1 as a novel biomarker for predicting patient prognosis and the effects of immunotherapy in diverse cancers. These results have been published as a preprint version [16], and our findings have potential to inform the future direction of research into TAP1.

## Methods and materials

### Clinical samples and ethics statement

Clinical glioblastoma (GBM) samples were obtained from inpatients at the Second Affiliated Hospital of Nanchang University between 2021 and 2022. Tumor core and para-tumor normal tissues were excised and stored at –80˚C until use. This study was approved by the Medical Ethics Committee of The Second Affiliated Hospital of Nanchang University. Each patient provided informed consent for sample acquisition and use for research based on the approved guidelines.

### Data sources and processing

mRNA expression profiles of *TAP1* in tumor and corresponding normal tissues were obtained from The Cancer Genome Atlas (TCGA) and Genotype-Tissue Expression (GTEx) databases. Available data were downloaded from the UCSC Xena database (https://xenabrowser.net/datapages/) [17], and the format changed to transcripts per kilobase million. A query of TPA1 in “TCGA pan-cancer atlas” was submitted via the cBioPortal website (https://www.cbioportal.org/). Data on gene alterations (mutation, structural variant, amplification, deep deletion, and multiple alterations) matched with 32 cancer types were obtained from “Cancer Type Summary” items. At the subcellular level, immunofluorescence images of TPA1 were obtained from The Human Protein Atlas (https://www.proteinatlas.org/) database and the subcellular distribution of TAP1 protein determined. Protein–protein interactions (PPI) were explored using the Compartmentalized Protein–Protein Interaction Database (https://comppi.linkgroup.hu/). Protein names were mapped using the “Retrieve/ID mapping” tool on the Uniprot website (https://www.uniprot.org/), and visualized using the R package “ggplot2” in the R programming environment. Cancer type abbreviations are summarized in Supplemental Table 1.

**Table 1:** The lymphocyte infiltration correlations with TAP1 expression

Cancer type	Activated CD4T memory	CD8T	B	Activated NK
BLCA	+	+	+	+
BRCA	+	+	+	+
CESC	+	+	+	+
COAD	+	+	Unclear	+
HNSC	+	+	Unclear	+
KIRC	Unclear	+	+	+
LIHC	+	+	+	+
OV	+	+	Unclear	+
READ	Unclear	Unclear	Unclear	Unclear
SKCM	+	+	+	+
STAD	+	+	Unclear	+
UCS	Unclear	Unclear	Unclear	Unclear

### Western blotting

Protein was extracted from collected GBM core and adjacent normal tissue samples, and prepared samples used to perform western blots using methods and reagents described previously [18]. The antibody used was rabbit TAP1 polyclonal antibody (Proteintech, number: 11114–1-AP; diluted 1:1000).

### Single-cell analysis of TAP1

*TAP1* single-cell analysis was conducted using the Tumor Immune Single-cell Hub (TISCH, http://tisch.comp-genomics.org/) website [19] Gene “TAP1” was input and cell-type annotation “major-lineage” searched in “all cancers”, to analyze *TAP1* expression in 33 cell types and 78 cancer lineages.

### Analysis of prognosis by Cox regression and Kaplan–Meier methods

The value of TAP1 for predicting patient prognosis in pan-cancer was explored based on matched *TAP1* expression and prognostic information from TCGA database. Univariate Cox regression and Kaplan–Meier analyses were performed to assess the relationships between *TAP1* expression and patient prognosis in diverse cancer types, using the outcome indices: overall survival (OS), disease-specific survival (DSS), disease-free interval (DFI), and progression-free interval (PFI). *TAP1* expression pattern was tested as a continuous variable by univariate Cox regression, and *TAP1* expression level as a bivariate variable using the Kaplan–Meier method. The algorithm, “surv-cutpoint” of the “surminer” R package was used to determine the cut-off point with maximal rank statistics that divided the undefined expression data into high- or low-expression sets. Given the non-normal distribution of survival data, a non-parametric test was applied and log-rank *P* values computed in Kaplan–Meier analysis. For Cox regression analysis, hazard ratio (HR) and 95% confidence interval (CI) values were calculated.

### Screening of differentially expressed genes (DEGs) between low and high TAP1 expression subgroups

Cancer patients were ordered according to their *TAP1* expression values and the 30% patient populations with the lowest and highest values defined as high and low expression subgroups, respectively. Differential expression analysis was performed using the “limma” R package [20], and log_2_(fold-change) and adjusted *P* values calculated. Genes with *P* < 0.05 were considered DEGs. DEGs for each cancer type are presented in Supplementary Table 2.

### Gene set enrichment analysis (GSEA)

GSEA was performed to further explore the possible mechanisms or biological processes involving TAP1. The Hallmark gene set (containing 50 gene sets) file was downloaded from MSigDB in “gmt” format. Then, the R package “clusterProfiler” used to conduct GSEA, based on data generated by differential expression analysis, with false discovery rate (FDR) and normalized enrichment score (NES) values computed for every hallmark in each cancer type [21]. TAP1 enrichment data in multiple pathways matched with corresponding pan-cancer types were visualized using the “ggplot2” R package.

### Pan-cancer tumor microenvironment analysis

Tumor masses are generally infiltrated by various immune cells and other functional cells that influence cancer progression and the effects of therapy, where infiltrating cells and molecules inside a tumor matrix comprise the tumor microenvironment (TME) [18]. Tumor Immune Estimation Resource 2.0 (TIMER 2.0) was used to quantitatively evaluate tumor immune cell infiltration based on transcriptome data from the pan-cancer cohort. Correlations between *TAP1* expression and infiltrating cells of interest were investigated using Spearman’s rank correlation analysis. Candidate cells included: CD4^+^ T cells, cancer-associated fibroblasts, lymphoid progenitors, myeloid progenitors, granulocyte-monocyte progenitors, endothelial cells, hematopoietic stem cells (HSCs), T cell follicular helper cells, T cell gamma delta cells, NK T cells, regulatory T cells, myeloid-derived suppressor cells (MDSCs), B cells, neutrophils, monocytes, macrophages, DC, NK cells, mast cells, and CD8^+^ T cells. Infiltration patterns were visualized using the R package “ggplot2”. Microsatellite instability (MSI) and tumor mutational burden (TMB) were evaluated as biomarkers to predict TME conditions [22, 23]. Correlations between *TAP1* mRNA expression and MSI or TMB were investigated by Spearman’s correlation test. According to a previous study, 47 immune checkpoints (ICP) were included and their correlations with *TAP1* expression also estimated [24].

### Immune check-point inhibitor (ICI) cohort validation

A comprehensive study to summarize the clinical effects of immune checkpoint blockade therapy was conducted. Clinical information, including immunotherapy prognosis, matched with *TPA1* expression data, were obtained from previous studies [25–28]. High or low expression was defined by the method applied in Kaplan–Meier survival analysis, and the prognosis of patients with different *TAP1* levels compared by log-rank test. To assess patient responses to ICIs, the chi-square test was applied to compare the proportions of patients that responded to ICI therapy.

### Statistical analysis

The Wilcoxon sum test was used to assess the statistical significance of differences in *TAP1* expression levels between cancer and para-cancerous normal tissues. A paired t-test was used to compare protein expression levels. To investigate correlations between cancer prognosis and *TAP1* expression, univariate Cox regression analysis and the Kaplan–Meier method were applied. Cox regression test, Cox *P* values, and HR were assessed, and log-rank *P* values with 95% CI calculated using the Kaplan–Meier method. Spearman’s correlation tests were employed to calculate the significance of correlations between *TAP1* expression and immune cell infiltration, immune regulator expression, TMB, and MSI. For immunotherapy cohort validation, differences in the proportions of responders and non-responders in low- and high- *TAP1* expression groups were evaluated by chi-square test. Statistical significance was set at *P* value < 0.05.

## Results

### Analysis of TAP1 at the genetic, mRNA, and protein levels

**Fig. 1:**
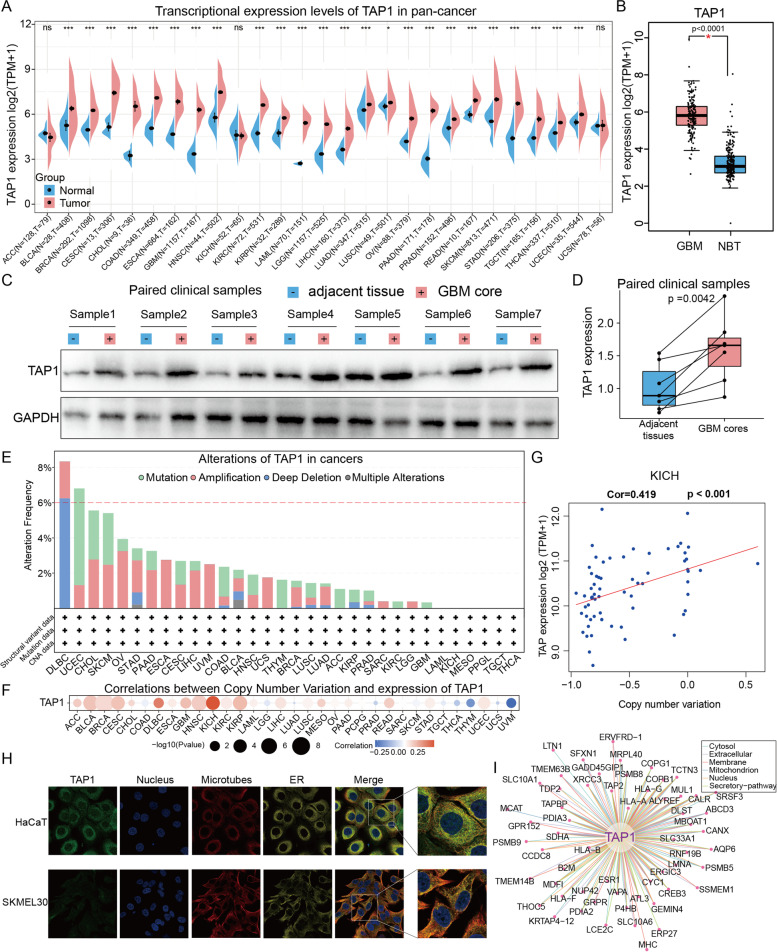
Analysis of TAP1 at the genetic, RNA, and protein levels. **A** RNA expression of *TAP1* in tumor and normal tissues from 27 pan-cancer types. **B** Comparison of differential *TAP1* RNA expression in GBM and NBT. **C** Comparison of TAP1 protein expression between cancerous and adjacent normal tissue from patients with GBM, the full-length blots/gels are presented in Supplemental Fig. 1. **D** Differences in TAP1 expression within paired clinical GBM samples (*n* = 7 pairs, *p* value = 0.0042). **E** Genomic alterations of *TAP1* across pan-cancer presented as types and frequencies of alterations. **F** Correlation between *TAP1* expression and copy number variation at the pan-cancer scale. **G** Analysis of correlation between copy number variation and *TAP1* expression level in KICH. **H** Distribution of TAP1 protein in the HaCaT and SKMEL30 cell lines; HaCaT is human non-malignant keratinocyte line and SKMEL30 is human melanoma cell line. **I** Protein–protein interactions of TAP1 in different cellular structures. GBM, glioblastoma; NBT, normal brain tissue; KICH, Kidney Chromophobe. *P < 0.05, **P < 0.01, ***P < 0.001

To determine the basic landscape of *TAP1* expression, multi-omics data on *TAP1* levels in various cancers were analyzed. Transcription profiles from TCGA and GTEx database were combined, as sample numbers for several cancer types were limited. *TAP1* mRNA expression in normal and tumor tissues from 27 cancer types is presented in Fig. 1A. *TAP1* was strongly significantly overexpressed in all tumor tissues, except adrenocortical carcinoma (ACC), kidney chromophobe (KICH), and uterine carcinosarcoma (UCS). Among tumors, *TAP1* showed marked overexpression in cervical and endocervical cancers (CESC) and HNSC, relative to other tumors, while the greatest difference between normal and corresponding malignant tissues was detected in cholangiocarcinoma (CHOL), GBM, and pancreatic adenocarcinoma (PAAD). GBM, the most malignant type of intracranial tumor, showed significantly higher *TAP1* expression relative to normal brain tissues (Fig. 1B). Overall, aberrant expression *TAP1* mRNA levels were detected in various cancer types. To determine whether TAP1 protein levels were also aberrantly expressed in cancer samples, we conducted western blot assays to verify the results of informatics analysis of RNA-seq data. As shown in Fig. 1C and ID, GBM samples from our patients also had higher TAP1 protein expression than corresponding adjacent normal tissues (*p* value = 0.0042), consistent with *TAP1* RNA expression patterns. Next, we investigated the genetic alteration status of *TAP1* in TCGA pan-cancer cohort, including the types and frequencies of genetic alterations (Fig. 1E). Among all alteration types, “amplification” was most frequent, followed by “mutation”, and “deep deletion”. Notably, some cancer types were observed to only have one type of *TAP1* genetic alteration; for example, esophageal adenocarcinoma and uveal melanoma (UVM) only exhibited amplification, while mutation was the sole alteration type in thymoma (THYM). In general, *TAP1* alteration frequencies in the pan-cancer cohort fluctuated between 2 and 4%; however, diffuse large B-cell lymphoma (DLBC) exhibited the highest frequency, at > 8%. Focusing on copy number variation in pan-cancer types, *TAP1* expression was significantly correlated with copy number variation in KICH and kidney renal papillary cell carcinoma (KIRP) (Fig. 1F). The scatter plot in Fig. 1G illustrates these results and a fitted regression curve.

Immunofluorescence images of cell lines derived from tumor and non-tumor tissue were analyzed to determine TAP1 protein distribution at the subcellular level (Fig. 1H). In both the para-cancerous normal (HaCaT) and melanoma (SKMEL30) cell lines, TAP1 protein was clearly located at the ER, with no change observed before and after tumorigenesis. Finally, we constructed a PPI network to identify potential biological interactions of TAP1 (Fig. 1I).

### Single-cell analysis of TAP1 expression results across multiple cell types

**Fig. 2:**
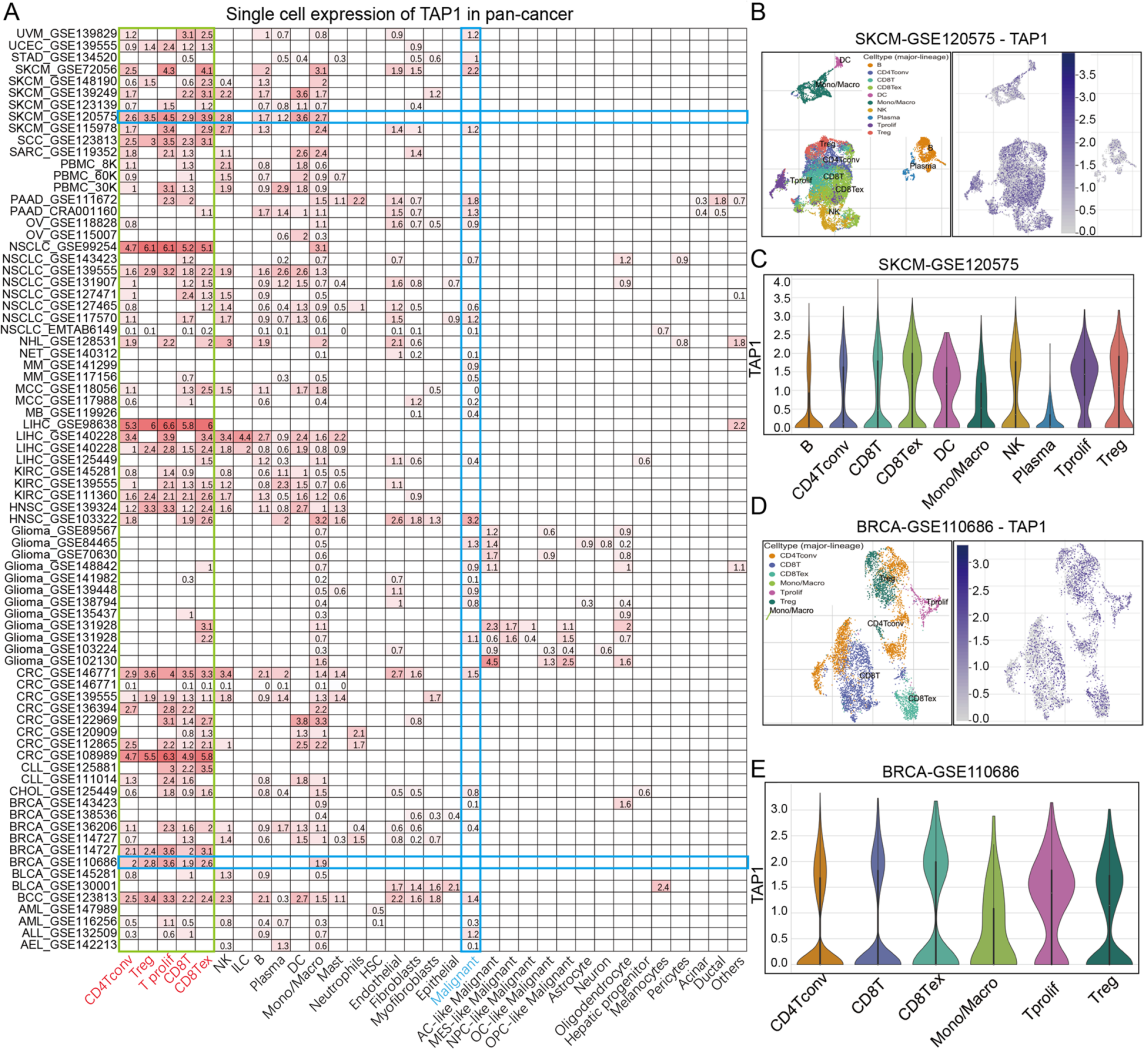
Single-cell scale TAP1 expression atlas in pan-cancer cohorts. **A** TAP1 expression values in each cell type from each cancer cohort. **B**, **C** Main cell types expressing TAP1 in the SKCM-GSE120575 cohort. **D, E** Main cell types expressing TAP1 in the BRCA-GSE110686 cohort

To determine *TAP1* expression patterns in tumor masses, we further explored the individual expression of *TAP1* in immune and malignant cell populations using the TISCH tool. *TAP1* expression was evaluated in all separate cells, and then presented as mean values (Fig. 2A). In the plotted heatmap, we observed that *TAP1* was mainly expressed in immune cells, particularly T lymphocytes (CD4 Tconv, T reg, T prolif, CD8 T, and CD8 Tex cells), followed by non-specific immune cells, including NK cells, DCs, and monocytes/macrophages. Notably, *TAP1* was not overexpressed in malignant cells, while it was strongly upregulated in cell populations of non-malignant origin. *TAP1* expression was stronger in the non-small cell lung cancer (GSE99254), liver hepatocellular carcinoma (LIHC; GSE98638), and CRC (GSE108989) datasets than in other cell lines. Specifically, we visualized *TAP1* expression in invasive breast carcinoma (BRCA; GSE11068) and skin cutaneous melanoma (SKCM; GSE12057) datasets, and the cell types with the highest *TAP1* expression are highlighted in Fig. 2B–E. Our results suggest preferential expression of *TAP1* in T lymphocytes and monocytes/macrophages in the TME.

### Risk prediction based on correlation between prognosis and TAP1 expression

**Fig. 3:**
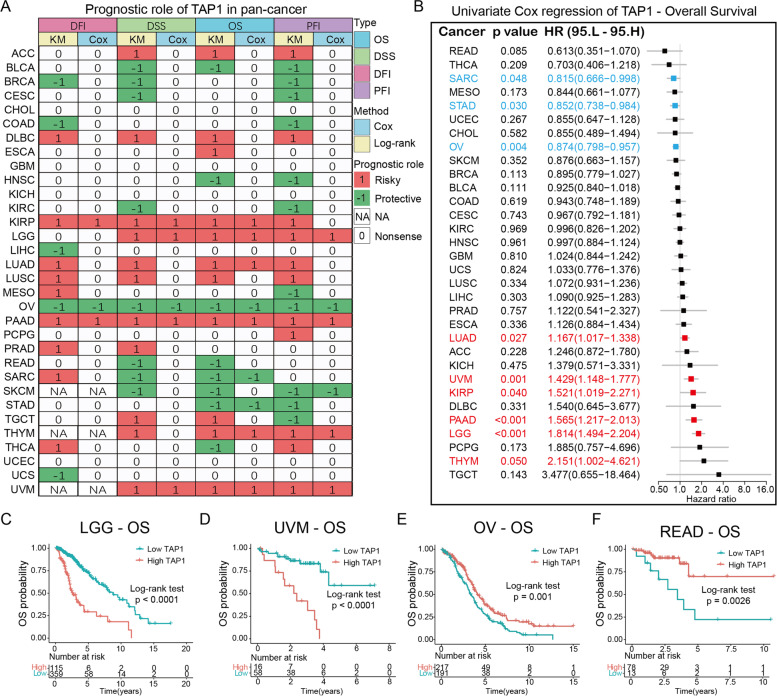
Correlation between TAP1 expression and cancer prognosis in pan-cancer cohorts. **A** Summary of the prognostic role of TAP1 in pan-cancers using Kaplan–Meier and Univariate Cox regression analyses. Clinical prognosis is expressed as DFI, DSS, OS, and PFI. **B** Forest plot showing cancer types correlated with TAP1 expression using OS data; HR and 95% CI are presented. Kaplan–Meier survival curve showing changes in OS probability against time in patients with low- and high- TAP1 expression in LGG (**C**), UVM (**D**), OV (**E**), and READ (**F**). DFI, disease-free interval; DSS, disease-specific survival; OS, overall survival; PFI, progression-free interval; HR, hazard ratio; CI, confidence interval; LGG, low-grade glioma; UVM, uveal melanoma; OV, ovarian serous cystadenocarcinoma; READ, rectum adenocarcinoma. The significance threshold was set as *P* < 0.05. *P < 0.05, **P < 0.01, ***P < 0.001

To further explore the potential value of TAP1 for prognosis prediction, we next analyzed the prognostic role of TAP1 across cancers. The survival indices, OS, DSS, DFI, and PFI, served as reliable indicators of prognosis. A summary of the clinical prognostic outcome patterns in the tested pan-cancer cohort are plotted in Fig. 3A. Kaplan–Meier and Cox regression analyses were performed to validate one another. The results suggested that TAP1 was a risk factor for patients with ACC, DLBC, KIRP, low-grade glioma (LGG), lung adenocarcinoma (LUAD), lung squamous cell carcinoma (LUSC), PAAD, and UVM, as higher expression of *TAP1* mRNA was correlated with poor prognosis, as well as a potential protective factor in bladder urothelial carcinoma (BLCA), BRCA, clear cell renal cell carcinoma (KIRC), ovarian serous cystadenocarcinoma (OV), rectum adenocarcinoma (READ), SKCM, stomach adenocarcinoma (STAD), and UCS. Cox regression analysis of OS data, revealed significant associations of *TAP1* expression in patients with sarcoma (SARC), STAD, OV, LUAD, UVM, KIRP, PAAD, LGG, and THYM (Forest plot, Fig. 3B). We conducted specific analyses of several cancer types and plotted Kaplan–Meier survival curves. As shown in Fig. 3C and D, OS rates of patients with LGG and UVM in the high-*TAP1* expression group decreased rapidly over time, while those in the low expression group had relatively better outcomes at the same time points. Clinical outcomes were most significantly reversed in patients with OV and READ (Fig. 3E–F). In some cancer types, including CHOL, esophageal carcinoma, GBM, KICH, paraganglioma, SARC, and UCS, there was no significant relationship between *TAP1* expression and OS. Cancer types showing significant correlations between *TAP1* expression and patient outcomes may benefit from TAP1-targeted treatment, which could be advantageous to populations sensitive to such regimens, as discussed below.

### TAP1 enriched hallmarks across the pan-cancer cohort

**Fig. 4:**
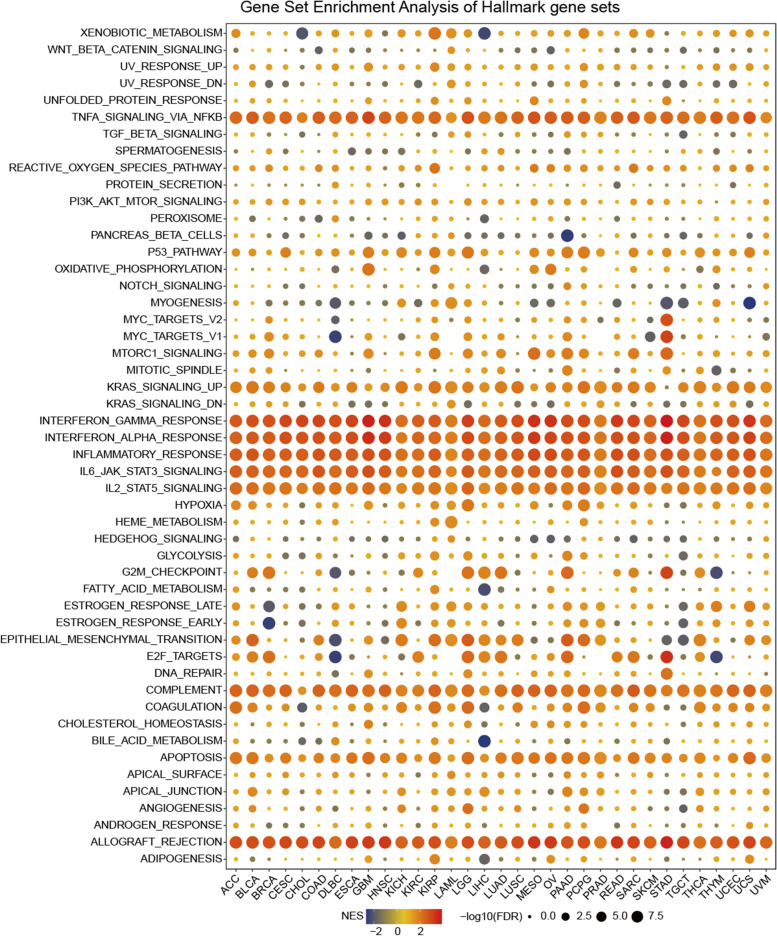
Analysis of correlations between *TAP1* expression level and enriched gene sets. Correlations between *TAP1* expression and enriched gene sets in pan-cancer displayed as a bubble plot of NES and log-rank FDR values. NES, normalized enrichment score; FDR, false discovery rate. Result were considered significant only when nominal *P* < 0.05 and FDR < 0.25

Given the significant prognostic value of TAP1 in various cancers, we further investigated the underlying biological processes or pathways associated with TAP1, to understand the potential mechanisms involved. In the present study, a hallmark gene set, composed of marker genes defining tumor biological status and progression, was analyzed. DEGs between high- and low-*TAP1* subgroups were screened and tested for enrichment in hallmarks gene sets. The enrichment status of TAP1 in each pathway is plotted in Fig. 4. Our results revealed a highly concentrated distribution of enrichment across 33 pan-cancer types, where immune-related pathways were strongly enriched for in cancers with high *TAP1* high expression. Enrichment pathways included tumor necrosis factor-α (TNF-α) signaling via the NF-κB pathway, interferon-γ (IFN-γ) response, IFN-α response, inflammatory response, IL6-JAK-STAT3 signaling pathway, IL2-STAT2 signaling pathway, and allograft rejection. TAP1 was also enriched in the apoptosis, complement, and KRAS signaling pathways, to a lesser degree. Focusing on cancer types, ACC, LGG, LUAD, PAAD, and UCS showed more enrichment of the pathways mentioned above. As illustrated in the bubble plot, the majority of enrichments exhibited positive correlations with *TAP1* expression (Fig. 4).

### Correlation between immune cell infiltration and TAP1 expression in pan-cancer

**Fig. 5:**
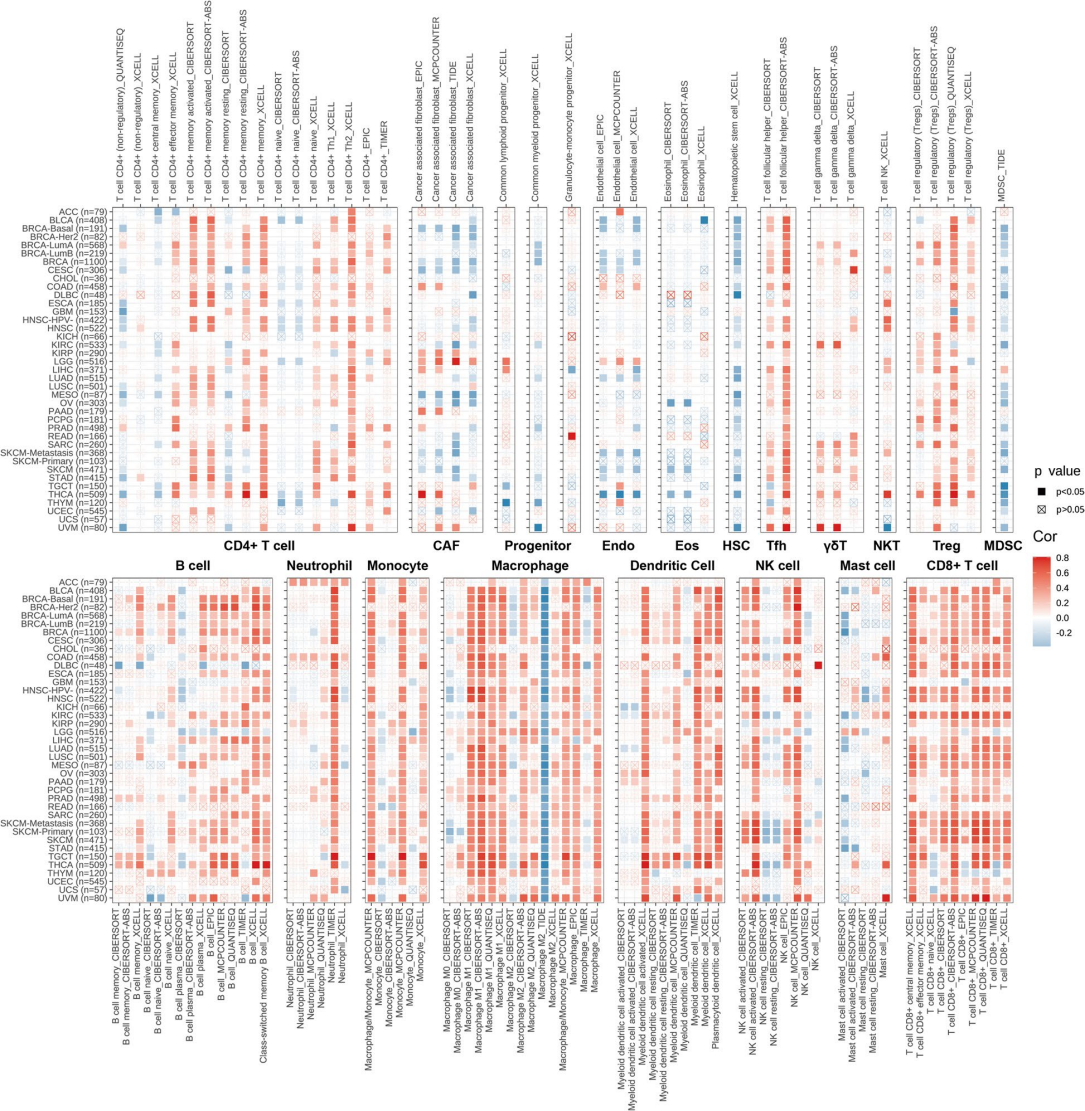
Correlation between *TAP1* expression and immune cell infiltration in various pan-cancer types. Correlations between *TAP1* expression and infiltration of 19 immune cell types were analyzed using TIMER 2.0. Spearman’s correlation analysis was applied to test significance. Red and blue blocks indicate positive and negative correlations, respectively. *P* < 0.05 was considered significant

Given the close correlation between *TAP1* expression and immune pathways, we next further explored its possible correlation with immune cell infiltration. Spearman correlation analysis of the relationship between *TAP1* expression and infiltration levels of multiple immune cell lineages in the pan-cancer cohort was conducted. The results presented in Fig. 5 illustrate the positive relationship between *TAP1* expression and several cell types, particularly macrophages, DCs, and CD8^+^ T cells. Further, positive correlations were mainly concentrated in the same cell lineages; that is, specific infiltrated cell types were positively correlated with *TAP1* expression in numerous cancer types. Overall, *TAP1* expression was positively correlated with the infiltration of most tested immune cells, other than some specific subtypes, such as HSCs, MDSCs, and M2 macrophages. Specifically, the majority of pan-cancer types were mainly infiltrated by CD8^+^ T cells, with the exceptions of ACC, CHOL, GBM, KICH, LGG, READ, and UCS. To link the prognostic role and single cell expression of *TAP1* in cancers, we speculated that the cancers with better prognosis and high *TAP1* expression also have high tumor infiltrating lymphocyte populations. We conclude that correlations between *TAP1* expression and four specific lymphocyte populations (activated CD4^+^ T, CD8^+^ T, B, and NK cells) infiltration levels were evident in 12 types of cancer in which TAP1 has a protective role. In accordance with expectations, in most of these cancers (BLCA, BRCA, CESC, colon adenocarcinoma (COAD), HNSC, KIRC, LIHC, OV, SKCM, and STAD, but not READ or UCS) *TAP1* levels were significantly correlated with lymphocyte infiltration (Table 1).

### Correlation between TAP1 expression and the TME

**Fig. 6:**
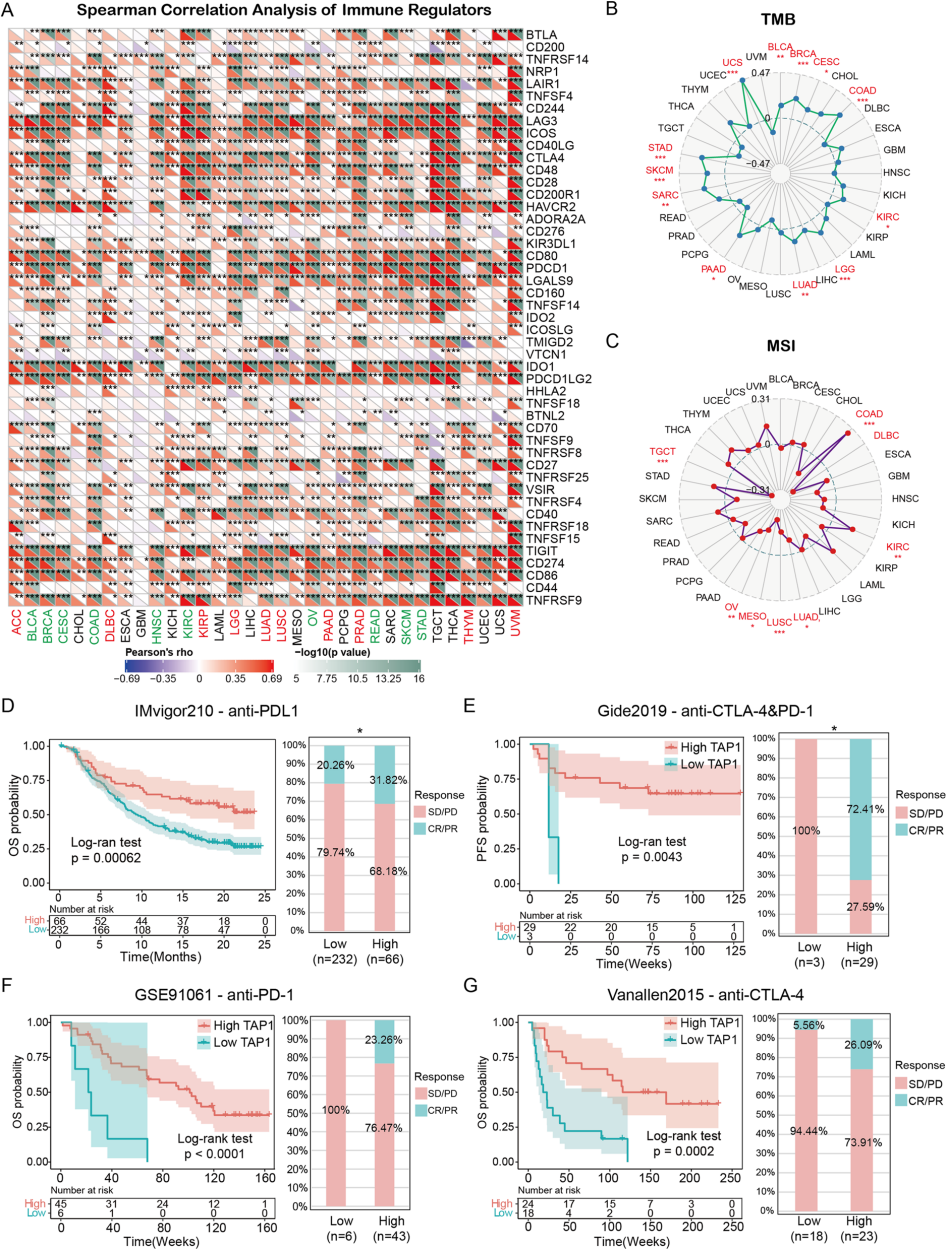
Correlations of *TAP1* expression with TME biomarkers and clinical responses to immunotherapy. **A** Heatmap showing correlations between *TAP1* expression and 47 immune regulators according to Spearman’s correlation test. **B**, **C** Correlations of *TAP1* expression with TMB and MSI. **D** Survival analysis of patients with high (*n* = 66) and low (*n* = 232) *TAP1* expression based on OS data from patients with urothelial cancer receiving anti-PDL1 immunotherapy, and proportions of patients with different therapeutic responses. **E** Survival analysis of patients with high (*n* = 29) and low (*n* = 3) *TAP1* expression based on PFS data from patients with melanoma receiving anti-CTLA-4&PD-1 immunotherapy, and the proportions of patients with different therapeutic responses. **F** Survival analysis of patients with high (*n* = 43) and low (*n* = 6) *TAP1* expression using OS data from patients with breast cancer receiving anti-PD-1 immunotherapy, and the proportions of patients with different therapeutic responses. **G** Survival analysis of patients with high (*n* = 23) and low (*n* = 18) *TAP1* expression using OS data from patients with metastatic melanoma receiving anti-CTLA-4 immunotherapy, and the proportions of patients with different therapeutic responses. TMB, tumor mutation burden; MSI, microsatellite instability; PD, progressive disease; SD, stable disease; CR, complete response; PR, partial responses. The significance threshold was *P* < 0.05. *P < 0.05, **P < 0.01, ***P < 0.001

Tumor cells generally evade immune attack by silencing immune responses. One strategy is to target and mute the immune regulators, so that functional immune signal processing is blocked, facilitating cancer cell survival. Here, 47 common ICP genes were selected and analyzed in the context of *TAP1* expression, using previously described methods [29]. Spearman’s correlation analysis was conducted to evaluate the correlations between *TAP1* expression and levels of individual ICPs across TCGA pan-cancer types (Fig. 6A). Overall, our results suggested a strongly significant positive correlation. Pan-cancer analysis indicated that *TAP1* expression was positively correlated with immune regulators in the majority of cancer types, particularly BRCA, KIRC, prostate adenocarcinoma (PRAD), testicular cancer (TGCT), thyroid carcinoma (THCA), and UVM. Regarding individual immune regulators, correlations with *TAP1* in each cancer were highly significantly positive or negative; specifically, LAG3, ICOS, HAVCR2, CD80, PDCD1, IDO1, PDCD1LG2, TIGIT, CD274, CD86, and TNFRSF9 exhibited markedly stronger correlations than other ICPs.

TMB is the quantity of acquired somatic mutations (after exclusion of innate mutations), which encode neoantigens, and serve as material for antigenic presentation. MSI represents an abnormal condition where numbers of repeated sequences are altered because of random insertion or deletion, suggesting an impaired DNA mismatch repair mechanism. Our analyses revealed correlations between *TAP1* expression and TMB (Fig. 6B), and MSI (Fig. 6C). Notable significant positive correlations of *TAP1* with TMB were detected in BLCA, BRCA, CESC, COAD, KIRC, LGG, LUAD, PAAD, SARC, STAD, and UCS, while MSI showed various degrees of significant positive correlation with *TAP1* expression in COAD, DLBC, KIRC, LUAD, LUSC, mesothelioma, OV, and TGCT.

### ICI cohort validation analysis

Laboratory findings always require confirmation and validation in clinical practice. Transcriptome profiles and clinical information, including OS and PFI and immunotherapy response data, from four cohorts in which patients with cancer received different immunotherapy regimens were obtained from published papers [25–28]. The immunotherapies applied were as follows: anti-programmed cell death protein 1 (PD-1), anti-programmed cell death protein 1 ligand (PDL1), and anti-CTL antigen 4 (CTLA4) treatment using monoclonal antibodies. As shown in Fig. 6D–G, groups with high *TAP1* expression had higher OS/PFI probability and longer OS/PFI time than those with low *TAP1* expression. Further, data on cancer therapeutic responses to immune therapy indicated that cohorts with melanoma or bladder cancer and high *TAP1* expression had a greater proportion of responders, indicating that patients with melanoma and bladder cancer and high *TAP1* levels had both worse clinical prognosis and were potentially more sensitive to ICI therapy.

## Discussion

At present, immunotherapy has been an efficient and promising treatment for cancer patients, and searching novel immune associated targets and biomarkers is urgent [30–32]. Byplotting of *TAP1* transcriptome data, derived from data mining, we clearly demonstrated that *TAP1* RNA levels were elevated in almost all tumor tissue types, other than ACC, KICH, and UCS; however, previous studies have reported down-regulation of both TAP1 mRNA and protein levels, inconsistent with our results [10–15]. Thus, it is reasonable to speculate that there may be genomic alterations that counteract the increased levels of *TAP1*. Genetic alteration analysis revealed a maximum frequency of *TAP1* gene alterations of 8% in the tested pan-cancer cohort, and mutation types were non-specific, indicating that they were unlikely to contribute substantially to cancer development. In addition, alterations in transcription may result in changes in TAP1 protein, and protein dysfunction can be reflected in changes of spatial distribution that influence function. In the present study, immunofluorescence images of melanoma and normal epithelial cells revealed that TAP1 was strictly distributed on the ER. Thus, the elevated expression of TAP1 could be attributed to neither specific types of genetic alteration or altered protein distribution. Hence, the reasons for aberrant *TAP1* expression in tumor tissue remain to be determined.

Generally, tumor tissue is composed of parenchyma and mesenchyme, and contains resident stromal cells and infiltrated immune cells, in addition to malignant cells. Hence, *TAP1* expression levels in tumor tissue represents a summation of that in all types of cells present in the tumor. As shown in Fig. 2A, *TAP1* expression was highly concentrated in various immune cells, particularly adaptive immune cells, including CD4^+^ and CD8^+^ T lymphocytes, followed by innate immune cells, such as monocytes/macrophages and DCs. Scattered *TAP1* expression was also detected among all candidate tumor cell lineages; however, levels were lower than those in immune cell lineages. The results of single cell analysis, may explain the contradictory findings between our transcriptional analysis and previous individual studies to some extent. Previous experiments indicating that TAP1 is downregulated in cancer cells were conducted at the cellular level, while our results were derived from a tissue-based analysis, with no separation of tumor cells from adjacent mesenchyme. Thus, the *TAP1* expression levels in all non-malignant cells were also quantified and may have led to detection of excess levels. Regardless, the detailed *TAP1* expression atlas generated here assists in understanding TAP1 distribution, providing a basis for further investigation.

We also focused on the clinical significance of TAP1, with the aim of informing its practical application. To evaluate the prognostic implications of *TAP1* expression, we used Kaplan–Meier and univariate Cox regression models to assess its clinical translational potential in each cancer type. Clinical prognostic outcomes were assessed using four indices: OS, DFI, DSS and PFI, each of which is characterized by a specific endpoint that reflects prognosis under different conditions. *TAP1* expression was detected as associated with both increased risk and protection, suggesting a distinct effect of TAP1 in each cancer. Forest plots of univariate Cox regression analysis of OS data indicated that the association of *TAP1* with survival probability varied among the 32 cancer types, with TAP1 a risk factor for 11 cancer types and a protective factor for 8 types. *TAP1* expression was positively correlated with OS of patients with BLCA, HNSC, OV, READ, SARC, SKCM, STAD, and THCA, indicating a protective role, while in 11 of 32 cancer types, including ACC, KIRP, LGG, LUAD, LUSC, PAAD, and UVM, *TAP1* expression was found to be a net risk factor. Specific Kaplan–Meier survival curve analysis of data from patients with LGG suggested that high *TAP1* expression was a risk factor for poor OS, while it was associated with opposite clinical outcomes in patients with BLCA, HNSC, and SKCM. Thus, TAP1 is a potentially promising and powerful prognostic biomarker for various cancers.

Given the significance of these results, we next sought to identify functional processes potentially involving TAP1. Using GSEA, we evaluated TAP1 enrichment in hallmarks gene sets, and found prominent enrichment in immune-related pathways, which was consistent across pan-cancer cohorts. TNF-α signaling, IFN-γ response, IFN-α response, inflammatory response, IL6-JAK-STAT3 signaling, IL2-STAT5 signaling, and allograft rejection were highly significantly enriched, with positive NES and low FDR values. IFN and TNF molecules promote MHC-I expression in vivo by inducing transcription activity [33, 34]. Further, IFN-γ and IFN-α/β have clear roles in TAP1 function, where IFN-γ can facilitate TAP-dependent peptide transport [35, 36]. Although MHC-I molecules and TAP are ubiquitously expressed in all nucleated cells at distinct levels, they are primarily expressed at sites of inflammation soon after immune system-mediated recognition and alert [37]. The results of our GSEA confirmed those of previously published papers. A strong correlation was observed between *TAP1* expression and pathways of interest. Allograft rejection, an immune rejection response against grafts from the same species, typically involve inflammatory responses of varying severity [38]. The most common form is acute rejection, which is mainly triggered by T cell-mediated immune responses [38]. Among interleukin-mediated signaling pathways, IL6 and IL2 are established inflammatory factors involved in tumor immunity regulation by facilitating lymphocyte growth and function [39, 40]. Overall, our results suggest an immune-related mechanism, which prompted us to further explore the potential of TAP1 to predict patient responses to immunotherapy.

Tumor development and progression rely on the adjacent TME, which comprises a complex variety of non-malignant cell types, including immune cells, fibroblasts, and endothelia, as well as extracellular components, such as cytokines and hormones [41]. Although the composition of the TME differs among cancers, all types have some common features. For example, in most tumors, the vascular network is relative leaky and disorganized, allowing infiltration of multiple immune cells for tumor immunity [42]. As we found that TAP1 was enriched in immune-related pathways, we also conducted an immune cell infiltration analysis to ascertain the associations between *TAP1* expression and infiltrated immune cells in the TME. Our results revealed an elaborate infiltration pattern, where *TAP1* expression was positively correlated with multiple immune cells, particularly CD8^+^ T cells, DCs, and macrophages. These results are consistent with those of our single-cell analysis, providing mutual verification, with CD8^+^ T cells and monocyte/macrophages, the killer cells of immune system, highlighted by both approaches. Among the numerous cell types analyzed by TIMER 2.0, M2 macrophages showed an opposite association, possibly because of the role of these cells in stimulating anti-inflammation, T helper 2 cell activation (assisting humoral immunity), and immunoregulation, which oppose the function of TAP1 in cell-mediated immunity [41]. Based on the results of single cell and prognostic analyses, we concluded that *TAP1* expression in lymphocytes has a role in cancers and that those with high *TAP1* expression are associated with better patient prognosis. High expression of *TAP1* in tumors is primarily in infiltrated lymphocytes, and indicates higher infiltration of lymphocytes, which is generally correlated with a stronger immune response, and could potentially explain the association of higher *TAP1* expression with superior patient prognosis.

Additionally, IL2-STAT5 signaling, inflammatory responses, and complement activity were highlighted in GSEA and are mediated by macrophages and CD8^+^ CTL. Combining currently available results, we conclude that TAP1 expression is highly correlated with immune regulation, and corresponds to a distinct immune signature for each pan-cancer type. Although abundant immune cells infiltrate tumors to mediate tumor immunity, the relationship between the TME and immune cells is complex. T cell-mediated tumor immunity can have either pro- or anti-tumor effects, depending on the cells and regulators they encounter during the process of immune responses [42]. In our study, 47 ICPs were tested for their correlation with *TAP1* mRNA expression across a pan-cancer dataset. Tumor cells adopt strategies to activate suppressive ICP pathways, thus silencing effector lymphocytes and evading immune surveillance [43]. Heatmap analysis indicated that *TAP1* expression was positively correlated with most ICPs in the majority of pan-cancer types, particularly BRCA, KIRC, PRAD, TGCT, THCA, and UVM. TMB and MSI are reported biomarkers that can predict TME status and anti-tumor efficacy of ICI therapy [44], and used Spearman’s correlation analysis to test the correlations of TMB and MSI with *TAP1* expression. The results highlighted specific cancers with significant associations. For example, both TMB and MSI were correlated with *TAP1* expression in COAD, KIRC, and LUAD. Hence, our results support the potential for *TAP1* expression to predict responses to immunotherapy targeting immune regulatory processes.

Precise therapy targeting tumor immunity based on distinct *TAP1* expression levels shows promise for application in cancer patients. Anti-tumor immunity is regulated by complex factors in the TME, including ICP, TMB, and MSI, and can generate different immune response outcomes [43]. PD-1, PD-L1, and CTLA-4 are established immunosuppressive ICPs, and are generally recruited by tumor cells to promote immune evasion [45, 46]. To date, monoclonal antibodies with high selectivity against PD-1 and CTLA-4 are approved and widely used in the clinic; however, the expected responses are only observed in a proportion of patients. As novel ICI therapies become popular, the lack of certainty that they will trigger a favorable response in specific individuals remain a problem. In cases where *TAP1* expression is highly correlated with immunotherapeutic biomarkers, it would be reasonable to expect the feasibility of immunotherapy for patients whose responses also correlate with *TAP1* expression. Information on clinical outcomes and transcriptome profiles of patients receiving immune therapy were also collected and analyzed. The results have potential to guide therapeutic decisions for patients. In previous studies, patients with primary or metastatic urothelial cancer, breast cancer, and melanoma were treated with single or combined monoclonal antibodies against PD-L1, PD-1, and CTLA-4, and the clinical outcomes suggest a protective role for TAP1 [25–28]. In our study, patients with BLCA, BRCA, and SKCM and high *TAP1* expression all exhibited better prognosis, consistent with the cohort analysis; h Especially, we also demonstrated that BLCA and SKCM cancer patients with higher TAP1 expression showed more sensitivity of ICI therapy in our study. We speculated that BLCA and SKCM patients with higher TAP1 expression might indicate higher lymphocyte infiltration in tumors, which usually results the tumor cells more vulnerable under the ICI therapy condition. owever, *TAP1* was not a favorable factor for immune therapy responses in all cancer types. As concluded based on the findings of prognostic and ICP correlation analyses, *TAP1* expression correlation varied among cancer types, which may be related to differences in the predictive role of TAP1. In LGG, high *TAP1* expression was associated with increased disease risk. Further, cancer may exhibit varied *TAP1* expression levels and clinical outcomes at different stages; for example, in stage 1 and 2 breast cancer, *TAP1* expression is reduced, while the trend is reversed in stage 3 and 4 disease; however, *TAP1* was considered a protective factor in our study [14]. Thus, we propose TAP1 as a promising and powerful biomarker to predict the effects of immunotherapy in patients with cancer. In addition to immunotherapy, previous studies have reported success in increasing tumor-specific immune responses by restoration of *TAP1* expression via a TAP1 expressing adenovirus [10]. Such novel treatments have inspired investigations of clinical prognosis and informed selection of optimal treatments based on the specific cancer types involved and individual transcriptome patterns of biomarkers, such as TAP1.

Although the present study provides rigorous evidence demonstrating the predictive role of TAP1 in clinical prognosis and potential responses to immunotherapy across pan-cancer, it has limitations. TAP1 is conventionally considered a tumor-associated gene; however, it showed diverse correlations with prognosis in pan-cancer analysis. Although we have proposed a possible explanation, a series of elaborate experiments are required to validate our hypothesis. Moreover, we proposed an essential role for TAP1 as predictor, but the practical clinical use of such an approach has not been verified. Furthermore, our investigation focused on population level analyses, whereas individual differences were neglected, and clinical therapy protocols are specific for individuals. These remaining issues indicate directions for future research, with the aim of providing advantages to patients requiring novel treatment for survival.

In conclusion, we conducted a systemic pan-cancer analysis with a novel design and characteristics. Our results revealed aberrant expression of *TAP1* in the majority of pan-cancer types, and that this expression is significantly correlated with clinical prognosis, immune cell infiltration, expression of ICPs, TME biomarkers, and immunotherapy efficacy.

Furthermore, we also clearly discuss our findings that were contradictory to those of previous studies, and hypothesize that *TAP1* expression in immune and stromal cells may have resulted in our finding that *TAP1* is upregulated tumor samples, which is not inconsistent with conclusions based on the role of TAP1 in samples comprising solely cancer cells. Hence, we propose TAP1 as a novel biomarker that can predict prognosis and immunotherapeutic responses in different cancer types, opening a new chapter in the exploration of TAP1 in malignancies.

## Conclusion

In this study, we conducted multi-omics research to explore the roles of TAP1 in prognostic prediction, immune cell infiltration, hallmarks associated with the TME, and prediction of immunotherapeutic responses on a pan-cancer scale. Our results indicate that TAP1 is a powerful and promising biomarker for predicting cancer prognosis and could benefit patients receiving immune therapy.

## Supplementary Information

**Additional file 1.**

**Additional file 2:** **Supplemental Table1.** Abbreviations of cancers in the TCGA-Pancancer cohort.

**Additional file 3:** **Supplementary Table 2.** The DEGs in each cancer type.

## Data Availability

This research recruited public databases and website tools. The data is available here: UCSC Xena: https://xenabrowser.net/datapages/. The supplementary materials can be found online. The original data and R codes can be obtained from the author (First Author, Zewei Tu, tuzewei@email.ncu.edu.cn) for reasonable requests.

## Abbreviations

ABC: ATP-binding cassette
ACC: Adrenocortical carcinoma
BLCA: Bladder urothelial carcinoma
BRCA: Breast invasive carcinoma
CAF: Cancer-associated fibroblast
CESC: Cervical and endocervical cancers
CHOL: Cholangiocarcinoma
CI: Confidence interval
ComPPI: Compartmentalized Protein Protein Interaction
COAD: Colon adenocarcinoma
CRC: Colorectal cancer
CTL: Cytotoxic T lymphocytes
CTLA-4: Cytotoxic T lymphocyte antigen 4
DC: Dendritic cell
DEG: Differential expression gene
DFI: Disease-free interval
DLBC: Diffuse large B-cell lymphoma
DSS: Disease-specific survival
EAC: Esophageal adenocarcinoma
ER: Endoplasmic reticulum
ESCA: Esophageal carcinoma
FDR: False discovery rate
GBM: Glioblastoma
GSEA: Gene set enrichment analysis
GTEx: Genotype-Tissue Expression
HPA: Human Protein Atlas
HNSC: Head and neck squamous cell carcinoma
HR: Hazard ratio
HSC: Hematopoietic stem cell
ICI: Immune check-point inhibitor
ICP: Immune checkpoints
IFN: Interferon
IL: Interleukin
KICH: Kidney Chromophobe
KIRP: Kidney renal papillary cell carcinoma
LGG: Low-grade glioma
LIHC: Liver hepatocellular carcinoma
LUAD: Lung adenocarcinoma
LUSC: Lung squamous cell carcinoma
MDSC: Myeloid-derived suppressor cell
MESO: Mesothelioma
MHC-I: Major histocompatibility complex class I
MSI: Microsatellite instability
NES: Normalized enrichment score
NSCLC: Non-small cell lung cancer
OS: Overall survival
OV: Ovarian serous cystadenocarcinoma
PAAD: Pancreatic adenocarcinoma
PCPG: Paraganglioma
PD-1: Programmed cell death protein 1
PD-L1: Programmed cell death protein 1 ligand
PFI: Progression-free interval
PPI: Protein–protein Interaction
PRAD: Prostate adenocarcinoma
READ: Rectum adenocarcinoma
SARC: Sarcoma
SCLC: Small cell lung cancer
SKCM: Skin cutaneous melanoma
STAD: Stomach adenocarcinoma
TAP1: Transporter associated antigen processing 1
TCGA: The Cancer Genome Atlas
Tfh: T cell follicular helper
TGCT: Testicular cancer
THCA: Thyroid carcinoma
TNF: Tumor necrosis factor
Treg: Regulatory T cell
γδT: T cell gamma delta
TIMER: Tumor Immune Estimation Resource
TISCH: Tumor Immune Single-cell Hub
THYM: Thymoma
TMB: Tumor mutational burden
TME: Tumor microenvironment
UCS: Uterine carcinosarcoma
UVM: Uveal melanoma
