# Supplementary figures and images for: Pan-cancer analysis: predictive role of TAP1 in cancer prognosis and response to immunotherapy
Source: BMC Cancer. 2023 Feb 9;23:133. doi: 10.1186/s12885-022-10491-w (PMC9912572; doi:10.1186/s12885-022-10491-w)

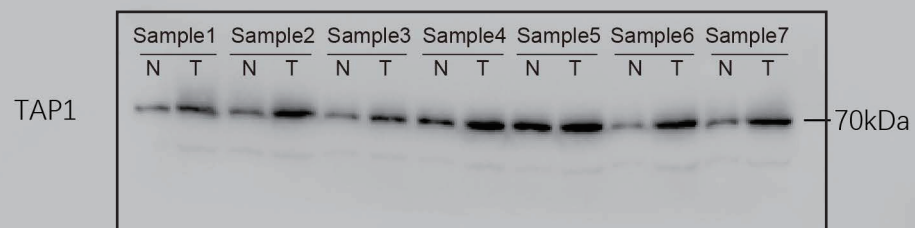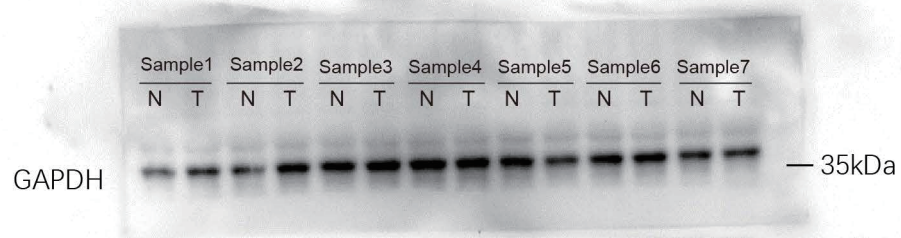

Supplement: Supplementary file 1 — Additional file 1. [file 12885_2022_10491_MOESM1_ESM.pdf]
